# Study of Mesoporous Zr-TiO_2_ Catalyst with Rich Oxygen Vacancies for N-Methylmorpholine Oxidation to N-Methylmorpholine-N-oxide

**DOI:** 10.3390/molecules29163812

**Published:** 2024-08-11

**Authors:** Yongwei Li, Zhihao Fang, Lijuan Feng, Fangfang Liu, Yucui Shi, Jiao Li, Chao Zhao

**Affiliations:** 1 School of Chemical Engineering and Environment, Weifang University of Science and Technology, Weifang 262700, China; wfsgfzh@163.com (Z.F.); ljfeng@alum.imr.ac.cn (L.F.); shiyucui1003@163.com (Y.S.); hglj23@126.com (J.L.); sdsgzhao@163.com (C.Z.); 2 Shandong Engineering Research Center of Green and High-Value Marine Fine Chemical, Weifang 262700, China

**Keywords:** Zr-TiO_2_, nanocatalyst, NMMO, selective oxidation

## Abstract

A series of Zr-TiO_2_ catalysts were prepared using a facile sol-gel method and were used for N-methylmorpholine (NMM) oxidation to N-methylmorpholine-N-oxide (NMMO). The structure features of Zr-TiO_2_ catalysts were studied in detail through a variety of characterization methods, such as XRD, SEM, N_2_ adsorption-desorption isotherms, XPS, EPR, and O_2_-TPD. As-obtained 5%Zr-TiO_2_ catalysts had superior catalytic performance and stability with a 97.6% NMMO yield at 40 °C, which related to Zr doping, a higher surface area, more oxygen vacancies, and oxygen chemisorption on the catalytic surface. This work provides an efficient preparation strategy of TiO_2_-based catalysts for selective oxidation reactions by a facile method.

## 1. Introduction

Cellulose, an inexhaustible renewable biomass resource in nature, has increasingly caused a great deal of concern in society [1,2,3]. N-methylmorpholine-N-oxide (NMMO) is a highly valuable solvent and a fine chemical for cellulose [4,5,6]. In recent years, most researchers have mainly focused on the application of NMMO in cellulose, with relatively little attention paid to the process of NMMO synthesis. The current method for producing NMMO commercially mainly involves the oxidation conversion reaction of N-methylmorpholine (NMM), in which the catalyst plays an important role [7,8]. Reported catalysts include bicarbonate [9], titanium silicon molecular sieves [10], etc. However, the traditional catalyst has some issues, such as a low catalytic efficiency, high reaction temperature, and many by-products. Therefore, it is still a crucial technological and scientific challenge to develop an efficient and gentle NMM catalytic oxidation system.

In recent years, research activities in the fields of nanoscience and nanotechnology have grown exponentially [11]. In metal oxide nanostructures, TiO_2_, one of the most popular research materials, is frequently utilized as a catalyst for selective oxidation because of its stability, weak acidity, environmentally friendliness, low cost, and surface properties [12,13,14]. However, the conversion of pure TiO_2_ may not be sufficiently ideal to meet the needs of commercial applications. The chemical and physical properties of titania can be modified by the doping of metallic ions [15]. The oxygen vacancy can be effectively adjusted by metal doping into TiO_2_ [16,17]. Nano-scale doping modifications can adjust the TiO_2_ surface active site, which is expected to further improve the activity of the catalyst while maintaining high selectivity [18]. Therefore, finding more affordable transition metals is a desirable option.

Among numerous doping elements, Zr has received widespread attention due to its unique electronic structure and chemical properties [19,20]. Zr has been used extensively in a variety of acid catalytic processes because of its Lewis acid characteristics under specific reaction circumstances [21,22]. Zr has excellent performance as a catalyst structure additive, can produce strong interactions with the active component, and easily produces oxygen vacancies, which is conducive to improving catalytic activity and selectivity [23,24]. Modifications of TiO_2_ with oxides of Zr are especially successful in controlling the structure-sorption characteristics of the composites. Zirconium is added during heat treatment at high temperatures to stabilize TiO_2_, inhibiting the formation of titania crystallites, and increasing the specific surface area [25,26]. Therefore, it is theoretically feasible to design Zr-TiO_2_ by doping Zr into a TiO_2_ substrate with good selectivity. Zhao et al. [27] synthesized a series of Zr-TiO_2_ catalysts, effectively converting ethyl levulinates to gvalerolactone using isopropanol as the H-donor. Kim et al. [28] reported the successful synthesis of Zr-doped TiO_2_ repeatedly using a solvothermal approach and its application on dye-sensitized solar cells, and achieved good results. These results indicate that metal Zr doping with TiO_2_ can effectively adjust the crystal structure and surface properties of TiO_2_, thereby significantly improving its catalytic performance. The study of Zr-TiO_2_ catalysts has important practical application value. However, there have been no reports on the application of Zr-TiO_2_ catalysts in NMMO synthesis.

Among the mature chemical synthesis methods, the sol-gel process is one of the common methods for preparing nanomaterials with a high surface area. This method has several advantages, including a simple process, low cost, good dispersion of dopants and titanium sources, and easy adjustments of the catalyst structure and size [29,30]. The emergence of new properties is ensured by mixing components at the molecular level. These are generally predictable and typically rely heavily on factors like the ratio of components, synthesis circumstances, etc. In this work, many materials with varied Zr contents of TiO_2_ were synthesized using a simple sol-gel technique to enhance the catalytic efficiency of the selective oxidation of NMM with H_2_O_2_. A series of characterization techniques were employed to gain a thorough understanding of the catalyst. To attain excellent catalytic performance, many reaction parameters and conditions were methodically tuned. A plausible reaction process for the synthesis of NMMO by NNM oxidation has been proposed, providing a promising method for producing NMMO under mild reaction conditions.

## 2. Results and Discussion

### 2.1. Structural Properties of the Catalysts

**Figure 1:**
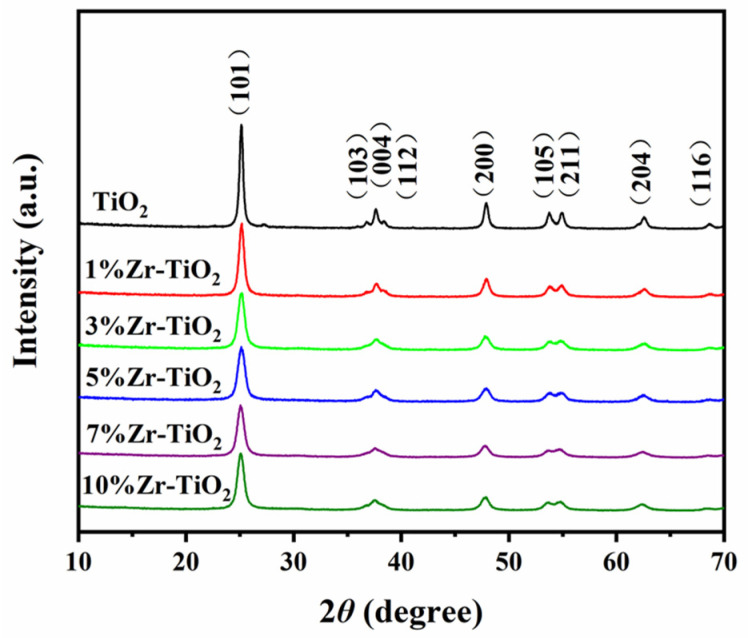
XRD pattern comparison of the catalysts.

Figure 1 presents the diffraction patterns of fresh TiO_2_ and Zr-TiO_2_. XRD spectra of TiO_2_ showed the presence of several peaks located at 25.1°, 36.7°, 37.6°, 38.4°, 47.9°, 53.7°, 54.9°, 62.5°, and 68.6°, corresponding to (101), (103), (004), (112), (200), (105), (211), (204), and (116) crystallographic planes of anatase, respectively, which are in agreement with the anatase TiO_2_ standard card (JCPDS No. 21-1272) [31]. Following zirconium doping, the XRD patterns of TiO_2_ resembled those of pure TiO_2_, and no peaks corresponding to zirconium oxides were seen. This suggests that the zirconium element may be present on the surface of the catalyst in the form of highly dispersed zirconia [32,33]. Zr^4+^ is more electropositive than Ti^4+^; therefore, each TiO_2_ nanoparticle may have a loosely held electron cloud, which promotes the creation of a less dense anatase phase [34]. Furthermore, the XRD patterns show that the positions of the XRD peaks did not change noticeably as the amount of zirconium doping increased. However, the fact that the Zr-TiO_2_ XRD peaks show considerable peak broadening in comparison to pure TiO_2_ suggests that the particle size of TiO_2_ doped with Zr element is smaller.

Table 1 displays the average particle size of the catalyst, which was calculated based on the (101) peak of the anatase using the Scherrer equation:

$$D=\frac{K\lambda }{\beta cos\theta }$$

where D is the average crystallite size of the catalyst, *λ* is the X-ray wave-length (1.54 Å), *β* is the full width at half maximum (FWHM) of the sample, *K* = 0.89, and *θ* is the diffraction angle. The average particle size of the catalyst decreases first and then increases weakly with increasing zirconium doping on TiO_2_ (Table 1). Therefore, the proper doping amount can prevent the growth and aggregation of TiO_2_ particles.

**Figure 2:**
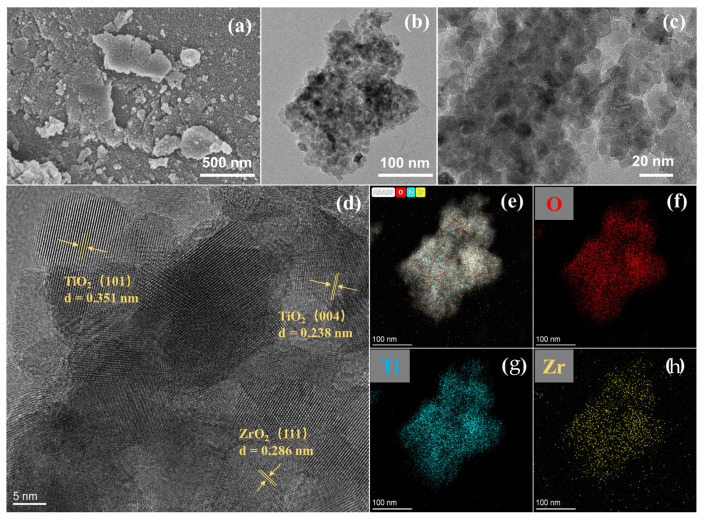
SEM (**a**), TEM (**b**–**d**), and EDS mapping of Zr, Ti, and O element (**e**–**h**) of 5%Zr-TiO_2_.

Next, SEM, TEM, and EDS studies were used to examine the morphology and elemental composition of the 5%Zr-TiO_2_ (Figure 2). SEM images (Figure 2a) revealed that the sample is composed of uniform small particles with no fixed morphology [35]. TEM images (Figure 2b,c) further confirmed that the 5%Zr-TiO_2_ powder was composed of nanocrystalline grains, which are evenly distributed with an average particle size of 10–20 nm. This result is consistent with the particle size calculated from XRD analysis data. Additionally, Figure 2d displays the particle distribution of different crystal surfaces. Two TiO_2_ crystal surfaces (101) and (004) with lattice values of 0.351 and 0.238 nm are observed [36]. Further, there is a crystal surface of d = 0.286 nm, which was assigned to ZrO_2_ (111), indicating that the doped Zr element in TiO_2_ exists mainly in the state of Zr oxide [37,38]. Moreover, EDS mapping (Figure 2e–h) showed the presence of O, Ti, and Zr as primary elements. This indicated that the doped Zr elements were uniformly distributed on the surface of the catalyst. These tests validated the successful integration of Zr species, and the efficacy of the synthetic methodology that was designed.

**Figure 3:**
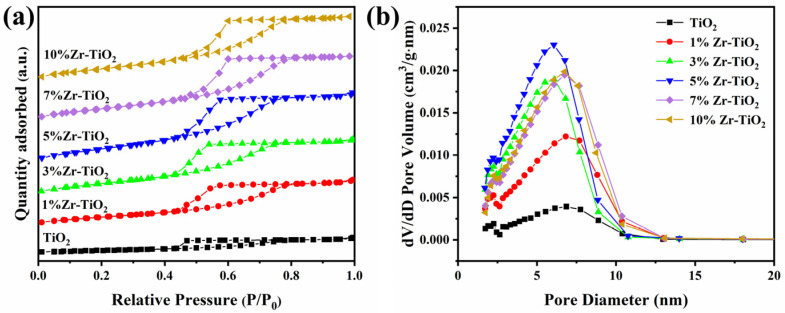
(**a**) N_2_ adsorption-desorption isothermal profiles and (**b**) pore size distribution of the catalysts.

As shown in Figure 3 and Table 1, the specific surface area and aperture information of catalysis were determined. Figure 3a shows that these catalysts have a hysteresis loop in the N_2_ adsorption-desorption isotherm. The isotherm displayed in all samples is a classic IV type, representing porous solids. The hysteresis loop indicates the presence of ink bottle or ink cage holes [15,39]. Moreover, most of the aperture distribution is concentrated in the 2–10 nm region (Figure 3b). Notably, Zr-TiO_2_ has a smaller pore size and larger specific surface area than pure TiO_2_ (Table 1). Additionally, a higher surface area was observed in the 5%Zr-TiO_2_ catalyst with a smaller crystal size. This can expose more active sites and improve the catalytic performance of NMM oxidation. The findings suggest that adding Zr to TiO_2_ species can modify the structural network to provide improved porous surfaces. This would increase the number of sites available for catalytic reactions and be advantageous for reactant molecule adsorption [40].

**Figure 4:**
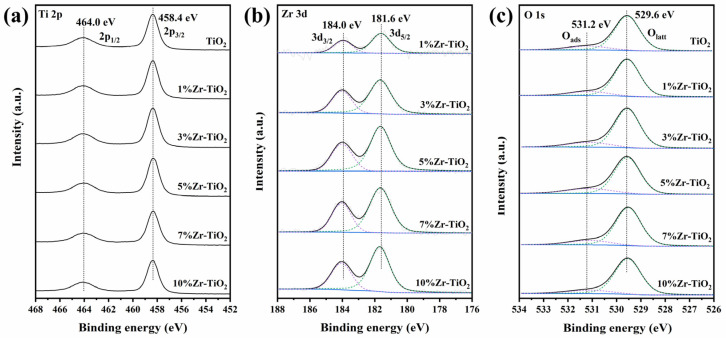
XPS spectra of all catalysts: (**a**) Ti 2p, (**b**) Zr 3d, and (**c**) O 1s.

XPS examination provided additional information about the chemical state and surface compositions of the catalysts (Figure 4 and Table 2). With reference to the C 1s peak position, which is fixed at 284.8 eV, the shift of the conventional peaks represented by solid curves was calibrated. The Zr element signal in the full spectrum of XPS gradually increased with the increase of Zr content (Figure S1). Two prominent peaks were observed in the XPS spectrum in the Ti 2p area for all catalysts in Figure 4a. These peaks are associated with the Ti 2p_3/2_ and Ti 2p_1/2_ signals of Ti species, and are situated at 458.4 and 464.0 eV, respectively, implying that Ti basically forms as Ti^4+^ [26,41]. Furthermore, two peaks were identified in the Zr 3d XPS region of the catalysts, which were linked to the Zr 3d_5/2_ and Zr 3d_3/2_ signals, respectively, and are positioned at 181.6 and 184.0 eV (Figure 4b). Furthermore, the separation of Zr 3d_5/2_ and Zr 3d_3/2_ signals was measured and found to be roughly 2.4 eV, which means that Zr exists chiefly in the Zr^4+^ state [42,43]. As the Zr content increased, there was a slight shift in the binding energy of Zr 3d towards a higher binding energy, which may be attributed to the interaction between Zr and Ti [44].

Furthermore, two distinct contributions were seen in the O1s XPS area (Figure 4c) at about 529.6 and 531.2 eV. These contributions are associated with the lattice oxygen (O_latt_) in bulk TiO_2_ and surface adsorbed oxygen (O_ads_), which was ascribed to Ti-O and surface -OH bonding [45,46]. With the increase of Zr content, the O_ads_ ratio of Zr-TiO_2_ first increased and then decreased. In contrast, Table 2 indicates that the 5%Zr-TiO_2_ catalyst has the highest proportion of O_ads_. This is likely due to the presence of more oxygen-defective sites, which have the tendency to interact with hydrogen atoms and generate more hydroxyl surface groups [47,48]. Moreover, Table 2 provides an overview of comprehensive information regarding element analysis. The Zr/Ti ratios of the surface are less than the Zr/Ti ratios predicted by theory, indicating an enrichment of Ti^4+^ on the catalyst surface. The O/Ti ratios of the surface are lower than the theoretical O/Ti ratios, indicating that the catalyst has surface defects and oxygen vacancies, which is helpful for improving catalytic qualities [49]. The C/Ti ratio of the sample surface is about 0.7–0.8, which may be attributed to the contact of the sample surface with carbon dioxide or other organic substances in the air, resulting in a carbon-containing signal in the test results.

**Figure 5:**
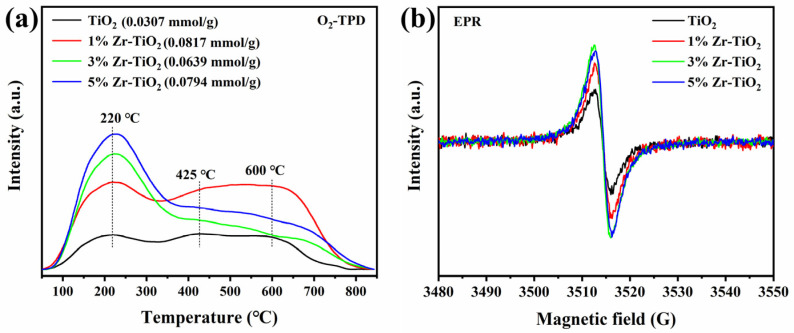
(**a**) O_2_-TPD spectrum and (**b**) EPR profiles of the catalysts.

O_2_-TPD was used to characterize the oxygen species of the catalysts, and the findings are displayed in Figure 5a. Within the temperature range of 0–800 °C, three different types of desorption peaks were detected in the sample. These peaks occur at approximately 220, 425, and 600 °C. Broadly speaking, there were three types of desorbed oxygen species found in the catalysts: adsorbed oxygen (O_2_^−^ and O^−^), lattice oxygen (O^2−^), and bulk lattice oxygen (O_bulk_^2−^) [50,51]. The surface of each catalyst is weakly linked to the chemically adsorbed oxygen species O_2_^−^ and O^−^, for which desorption took place between 150 and 350 °C. The desorption temperature range for the surface O^2−^ was between 350 and 670 °C, which is higher than the bulk lattice oxygen’s desorption temperature of 700 °C [52]. Since surface lattice oxygen desorbs at a temperature over 350 °C, the catalyst’s peak below 350 °C represents the desorption of chemically adsorbed oxygen [53,54]. NMM typically oxidizes at temperatures lower than 100 °C, with surface adsorbed oxygen taking part in the catalytic oxidation reaction. Figure 5a shows that the Zr-TiO_2_ catalysts have a much larger O_2_ desorption capacity than the pure TiO_2_ catalyst, particularly the 5%Zr-TiO_2_ catalyst. This suggests that adding Zr to the catalyst increases the number of active oxygen species. To further study the oxygen vacancy signal on the catalyst surface, oxygen vacancy measurements were recorded by paramagnetic resonance spectrometer (Figure 5b). As the Zr content increased, the oxygen vacancy signal was obviously enhanced. By maximizing the reactant adsorption energy on the catalyst surface, oxygen vacancies can lower the reaction energy barrier and encourage molecular activation. In the catalyst, oxygen vacancies collaborate with adjacent active metal sites in a beneficial manner [55]. As a result, the addition of Zr species accelerated the oxidation of NMM by creating more oxygen vacancies and surface oxygen.

### 2.2. Catalytic Performance

In our preliminary research, the metal oxides CuO, MgO, ZnO, Al_2_O_3_, ZrO_2_, TiO_2_, and SiO_2_ were proven to be active for NMM oxidation (Figure S2). As expected, we found that TiO_2_ exhibits high catalytic performance in the oxidation of NMM to NMMO, with excellent NMMO selectivity. Furthermore, ZrO_2_ shows slightly better conversion and slightly lower selectivity than TiO_2_.

**Figure 6:**
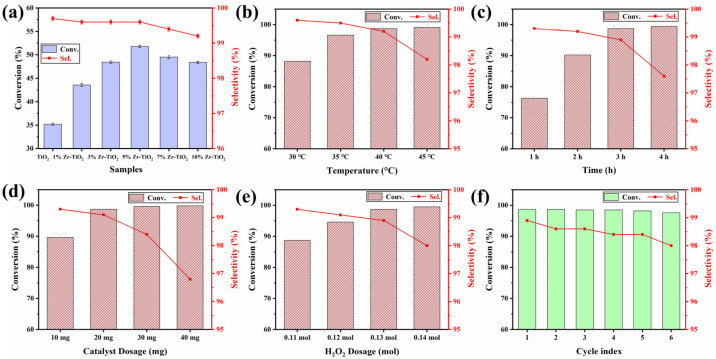
(**a**) Catalyst screening. Conditions: 0.1 mol NMM, 0.13 mol H_2_O_2_, 20 mg catalysts, 30 °C, 0.5 h. (**b**) Effect of temperature. Conditions: 0.1 mol NMM, 0.13 mol H_2_O_2_, 20 mg 5%Zr-TiO_2_, 3 h. (**c**) Effect of time. Conditions: 0.1 mol NMM, 0.13 mol H_2_O_2_, 20 mg 5%Zr-TiO_2_, 40 °C. (**d**) Effect of catalyst dosage. Conditions: 0.1 mol NMM, 0.13 mol H_2_O_2_, 40 °C, 3 h. (**e**) Effect of H_2_O_2_ dosage. Conditions: 0.1 mol NMM, 20 mg 5%Zr-TiO_2_, 40 °C, 3 h. (**f**) Cycling stability of 5%Zr-TiO_2_.

In this study, to compare the performance of the prepared catalyst, catalyst activity screening experiments were carried out by magnetic stirring in a three-neck flask at 30 °C for 0.5 h. The prepared catalyst was tested during the oxidation process of NMM by H_2_O_2_, and the results are shown in Figure 6a. The statistical results of these repeated experiments are summarized in Table S1.

As can be seen from Figure 6a, pure TiO_2_ provided only 35.5% NMM conversion and approximately 99.8% NMMO selectivity. As expected, when the Zr content in TiO_2_ increased from 1% to 10%, the NMM conversion first increased and then decreased, with 5%Zr-TiO_2_ showing the highest conversion rate (51.4%). Under the same circumstances, the NMMO selectivity showed a weak trend of continuously decreasing. This suggested that a key factor in the catalytic oxidation process was the interaction between the appropriate amount of Zr and Ti structures. Clearly, 5%Zr-TiO_2_ showed a slightly higher NMMO yield. Consequently, 5%Zr-TiO_2_ was determined to be the ideal catalyst for additional tuning based on NMMO yields.

Figure 6b–e illustrates the results of a study conducted on the impacts of reaction parameters, including temperature, reaction duration, catalyst dosage, and H_2_O_2_ dosage. Increasing the temperature led to higher conversions, but a temperature above 40 °C caused a noticeable decrease in NMMO yield due to reduced selectivity (Figure 6b). A possible explanation for this result is that high temperature is conductive to the decomposing of H_2_O_2_ into free radicals, which results in the formation of by-products [56]. Therefore, the active oxygen in H_2_O_2_ is not utilized effectively under high temperatures. Increasing the reaction time gradually increased the conversion, but when the reaction time exceeded 3 h, the selectivity decreased significantly (Figure 6c). The NMM conversion rose as the catalyst dosage increased from 10 mg to 40 mg, although the NMMO yield first climbed and subsequently somewhat dropped due to a decrease in selectivity (Figure 6d). Therefore, the optimal catalyst dosage is 20 mg. In a similar manner, the oxidizability of NMM was investigated at various molar ratios of H_2_O_2_/NMM (Figure 6e). A high molar ratio of H_2_O_2_ to substrate is advantageous for selective oxidation within a certain range. Increased H_2_O_2_ dosages result in increased conversions; however, if the dose is greater than 0.13 mol, the over-oxidation of NMM causes a noticeable drop in yield. Therefore, the optimum H_2_O_2_ dosage is 0.13 mol. Based on the previously provided information, the optimal reaction conditions were determined to be 0.1 mol NMM, 0.13 mol H_2_O_2_, 20 mg 5%Zr-TiO_2_, 40 °C, and 3 h. Under optimum reaction circumstances, the conversion of NMM and the selectivity of NMMO can approach 98.7% and 98.9%, respectively, even with a minimal amount of catalyst.

In terms of cost-effective and eco-friendly procedures, the durability and recyclability of catalysts are essential elements. Following the reaction, the catalyst was filtered; cleaned three times with ethanol, water, and dichloromethane; and then dried for 3 h at 80 °C. After the sixth run, the NMM conversion barely decreased to 97.6%, with a slight decrease in selectivity (Figure 6f). The findings showed that, under ideal circumstances, the 5%Zr-TiO_2_ combination was very stable in this investigation. A comparison between the performance of the catalyst prepared in this study and the traditional catalyst is summarized in Table S2.

### 2.3. Reaction Process

The XRD and TEM data demonstrated that the sol-gel approach is an efficient means to create Zr-TiO_2_ catalysts with a variety of crystal sizes. The Zr content of these catalysts can be adjusted to alter their crystal sizes. Smaller crystal diameters are typically the consequence of increased Zr concentrations. For catalysts with smaller crystal sizes, the N_2_ adsorption-desorption isotherms show a greater surface area. Zr doping increases the amount of adsorbed oxygen and improves surface active sites, as confirmed by XPS analysis, which also shows the presence of surface defects and oxygen vacancies. The results of O_2_-TPR and EPR tests indicate that the 5%Zr-TiO_2_ catalyst with more adsorbed oxygen and oxygen vacancies has superior catalytic oxidation, producing more active oxygen species and increasing the catalytic oxidation of NMM.

**Scheme 1:**
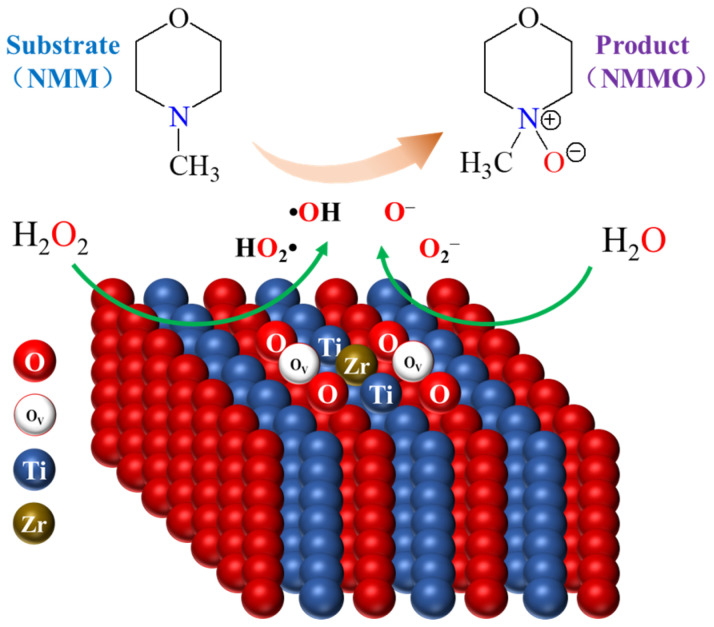
The schematic diagram of the catalytic oxidation process.

Based on the above data and free radical test analysis (Figure S3), a plausible reaction process for the selective catalytic oxidation of NMM to NMMO by Zr-TiO_2_ with H_2_O_2_ was delineated in Scheme 1. The catalytic activity of Zr-TiO_2_ is enhanced by its large specific surface area structure and plentiful surface oxygen vacancies. First, some H_2_O_2_ is adsorbed onto the catalyst surface for activation, forming a metal peroxide species [57] that improves H_2_O_2_ utilization efficiency by selectively oxidizing NMM to NMMO. In the meantime, activation of H_2_O_2_ produces strong oxidizing hydroxyl radicals (•OH) and peroxide radicals (HO_2_•) [58], which function as active oxygen species to oxidize NMM to NMMO with minimal by-products. Additionally, reactive oxygen species (O_2_^−^ and O^−^) are formed by the catalyst’s surface chemical adsorption activation [51], which oxidizes NMM to NMMO and speeds up the oxidation of the substrate NMM.

## 3. Experimental Section

### 3.1. Chemical Reagents

Butyl titanate, zirconium nitrate pentahydrate, anhydrous ethanol, glacial acetic acid, and dichloromethane were purchased from Aladdin Biochemical Technology Co., Ltd. (Shanghai, China). Every chemical used was of p.a. quality, and no further purification was necessary.

### 3.2. Zr-TiO_2_ Catalyst Preparation

A series of Zr-TiO_2_ nanoparticles were prepared by the sol-gel method. Solution A was prepared by adding 17 g (50 mmol) of butyl titanate dropwise to 20 mL of anhydrous ethanol and stirring for 30 min. Solution B was prepared by dissolving zirconium nitrate pentahydrate (*x* mmol) in a homogeneous solution of anhydrous ethanol (10 mL), glacial acetic acid (4 mL), and deionized water (2 mL) and stirring for 30 min. Then, solution B was added dropwise to solution A. The pH of the solution was adjusted to 2 using acetic acid and stirred for 6 h to form a sol. Subsequently, sonication was performed for 30 min, followed by aging at 50 °C for 12 h. Zr-TiO_2_ nanopowder was obtained through vacuum drying and calcination at 500 °C for 3 h. The samples with different amounts of zirconium nitrate pentahydrate added (*x* = 0, 0.5, 1.5, 2.5, 3.5, 5.0 mmol) were denoted as TiO_2_, 1%Zr-TiO_2_, 3%Zr-TiO_2_, 5%Zr-TiO_2_, 7%Zr-TiO_2_, and 10%Zr-TiO_2_.

### 3.3. Characterization

Powder XRD (D8 Advance A25, Bruker, Bremen, Germany) was used to analyze the crystal structure and arrangement of the sample throughout a 2θ range of 10° to 80°. The morphology and the microstructure of the Zr-TiO_2_ nanopowder were examined by SEM (MIRA LMS, TESCAN, Brno, Czech Republic) and TEM (Talos F200X G2, FEI, Waltham, MA, USA). The surface chemical composition and elemental state were analyzed by X-ray photoelectron spectroscopy (Scientific K-Alpha, Thermo, Waltham, MA, USA). The specific surface area information was measured by a N_2_ adsorption and desorption isotherm instrument (ASAP 2460, Micromeritics, Atlanta, GA, USA). The specific surface area was determined by the Brunauer–Emmett–Teller (BET) method and the pore size was obtained by the Barrett–Joyner–Halenda (BJH) method. O_2_ temperature-programmed desorption was performed under a chemisorption analyzer (5080B, xianquan, Tianjin, China). Free radical and oxygen vacancy measurements were recorded by paramagnetic resonance spectrometer (EMXnano, Bruker, Bremen, Germany).

### 3.4. Catalytic Activity Test

Quantitative NMM (0.1 mol) and a trace amount of catalyst (20 mg) were added to a three-necked jacketed round bottom flask for the NMM oxidation reaction. At 30 °C, 0.13 mol H_2_O_2_ was gradually added to the NMM/catalyst mixture while being stirred by magnetic means. Following the injection of H_2_O_2_, the reaction system was maintained at 30 °C. Reaction product samples were taken every 0.5 h for HPLC (Flexar, PerkinElmer, MC, USA) analysis to ascertain the contents of NMM and NMMO. During the test, morpholine (M) and nitroso-morpholine (NMOR) were mainly considered as possible by-products in the reaction system. Based on this, standard curves were established using standard samples of M, NMM, NMMO, and NMOR (Figure S4).

The conversion and selectivity were calculated according to Equations (1) and (2):

(1) $$Conversion\;(\%)=\frac{{C(NMM)}_{Initial}-{C(NMM)}_{Final}}{{C(NMM)}_{Initial}}\times 100$$

(2) $$Selectivity\;(\%)=\frac{C(NMMO)}{{C(NMM)}_{Initial}-{C(NMM)}_{Final}}\times 100$$

where *C(NMM)* and *C(NMMO)* represent the concentrations of *NMM* and product *NMMO* (mol/mL). Throughout the studies, it was expected that the system’s overall volume would not change. The conversion and selectivity figures were all given as molar percentages.

## 4. Conclusions

In a word, a series of efficient Zr-TiO_2_ catalysts were prepared by a facile sol-gel method and applied to NMM oxidation to NMMO. An appropriate quantity of the transition metal Zr can be added to the catalyst to effectively increase its specific surface area and the contact area of multiphase catalytic processes, according to experiments and characterizations. Simultaneously, the addition of Zr considerably raises the oxygen vacancy concentration and oxygen chemisorption on the catalytic surface, which greatly increases the activity of the catalyst. The catalytic activity experiment indicated that 5%Zr-TiO_2_ exhibited the best catalytic performance, and the optimized conditions for the reaction were determined to be 0.1 mol NMM, 0.13 mol H_2_O_2_, 20 mg 5%Zr-TiO_2_, 40 °C, and 3 h. Even with a small amount of catalyst, the conversion of NMM and yield of NMMO reached 98.7% and 97.6%, respectively. In addition, cyclic experiments showed that 5%Zr-TiO_2_ catalysts have good stability. In conclusion, this study offered a new strategy for the design of highly efficient catalysts in NMMO industrial production.

## Figures and Tables

**Table 1:** The crystal size, surface area, and pore diameter of the catalysts.

Catalysts	Crystal Size (nm)	Surface Area (m^2^/g)	Pore Diameter (nm)
TiO_2_	21.7	16	6.1
1%Zr-TiO_2_	14.6	49	5.7
3%Zr-TiO_2_	11.5	72	5.1
5%Zr-TiO_2_	10.6	85	5.0
7%Zr-TiO_2_	11.1	75	5.4
10%Zr-TiO_2_	11.4	74	5.5

**Table 2:** The surface compositions of all catalysts.

Catalysts	[Zr]/[Ti]	[O]/[Ti]	[C]/[Ti]	O_ads_/(O_ads_ + O_latt_)%
TiO_2_	--	1.98	1.00	12.6
1%Zr-TiO_2_	0.007	1.99	0.72	13.5
3%Zr-TiO_2_	0.015	2.05	0.78	15.0
5%Zr-TiO_2_	0.021	2.06	0.75	18.3
7%Zr-TiO_2_	0.030	2.04	0.77	15.4
10%Zr-TiO_2_	0.045	2.15	0.72	14.8

## Data Availability

The original contributions presented in the study are included in the article/Supplementary Materials, further inquiries can be directed to the corresponding authors.

## Supplementary Materials

The following supporting information can be downloaded at: https://www.mdpi.com/article/10.3390/molecules29163812/s1. Figure S1: XPS full spectra of the catalysts. Figure S2: Performance comparison of different catalysts. Conditions: 0.1 mol NMM, 0.13 mol H_2_O_2_, 20 mg catalysts, 30 °C, 0.5 h. Table S1: Effect of different catalysts on NMM oxidation by H_2_O_2_. Figure S3: EPR spectra of 5%Zr-TiO_2_. Figure S4: HPLC standard curve. Table S2: Comparison with reported catalyst properties.
