# Supplementary material for: Study of Mesoporous Zr-TiO2 Catalyst with Rich Oxygen Vacancies for N-Methylmorpholine Oxidation to N-Methylmorpholine-N-oxide
Source: Molecules. 2024 Aug 11;29(16):3812. doi: 10.3390/molecules29163812 (PMC11357547; doi:10.3390/molecules29163812)
Supplement: Supplementary file 1 [file molecules-29-03812-s001.zip › molecules-3136084-supplementary.pdf]

Nanocatalysis Zr-TiO2: An efficient catalyst for the synthesis of N-methylmorpholine-N-oxide under mild conditions

Yongwei Li [1,2,*], Zhihao Fang [1,2], Lijuan Feng [1,2], Fangfang Liu [1,2,*], Yucui Shi [1,2], Jiao Li [1] and Chao Zhao [1]

1 School of Chemical Engineering and Environment, Weifang University of Science and Technology, Weifang 262700, China; wfsgfzh@163.com (Z.F.); ljfeng@alum.imr.ac.cn (L.F.); shiyucui1003@163.com (Y.S.); hglj23@126.com (J.L.); sdsgzhao@163.com (C.Z.);

2 Shandong Engineering Research Center of Green and High-value Marine Fine Chemical, Weifang 262700, China

*Correspondence: liyongwei@wfust.edu.cn (Y.L.); liuff10507@wfust.edu.cn (F.L.);

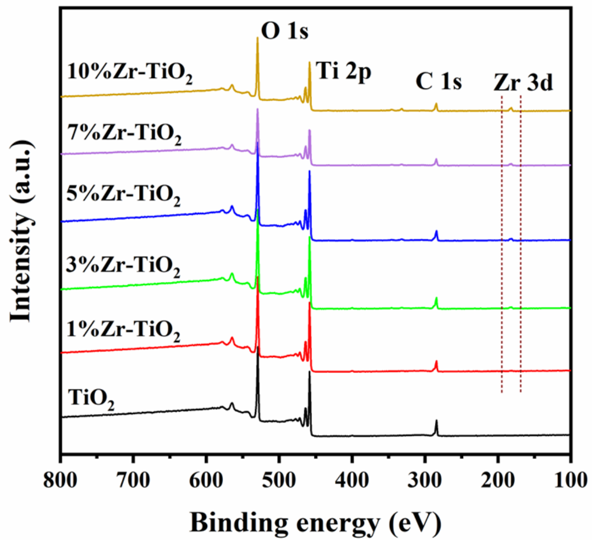

Figure S1. XPS full spectrum of the catalysts.

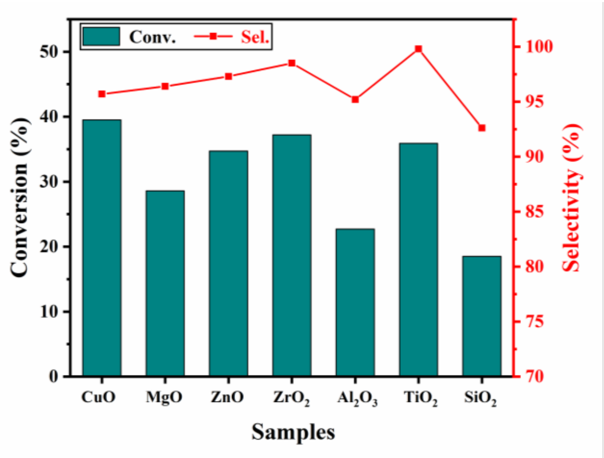

Figure S2. Performance comparison of different catalysts. Condition: 0.1 mol NMM, 0.13 mol H_2O_2, 20 mg catalysts, 30 °C, 0.5 h.

Table S1. Effect of different catalysts on NMM oxidation by H_2O_2.

Entry	Catalyst	Conversion （%）						Selectivity （%）					
		1st	2nd	3rd	Mean	S.D.	S.E.	1st	2nd	3rd	Mean	S.D.	S.E.
1	TiO_2	35.2	34.8	35.6	35.2	0.4	0.2	99.8	99.6	99.7	99.7	0.1	0.1
2	1%Zr-TiO_2	43.2	43.4	44.1	43.6	0.5	0.3	99.6	99.7	99.5	99.6	0.1	0.1
3	3%Zr-TiO_2	48.4	48.0	48.9	48.4	0.5	0.3	99.7	99.6	99.5	99.6	0.1	0.1
4	5%Zr-TiO_2	51.7	51.4	52.3	51.8	0.5	0.3	99.7	99.6	99.5	99.6	0.1	0.1
5	7%Zr-TiO_2	49.3	49.1	50.2	49.5	0.6	0.3	99.4	99.5	99.3	99.4	0.1	0.1
6	10%Zr-TiO_2	48.4	48.0	48.8	48.4	0.4	0.2	99.2	99.1	99.3	99.2	0.1	0.1

Notes: Standard Deviation(S.D.), Standard Error(S.E.).

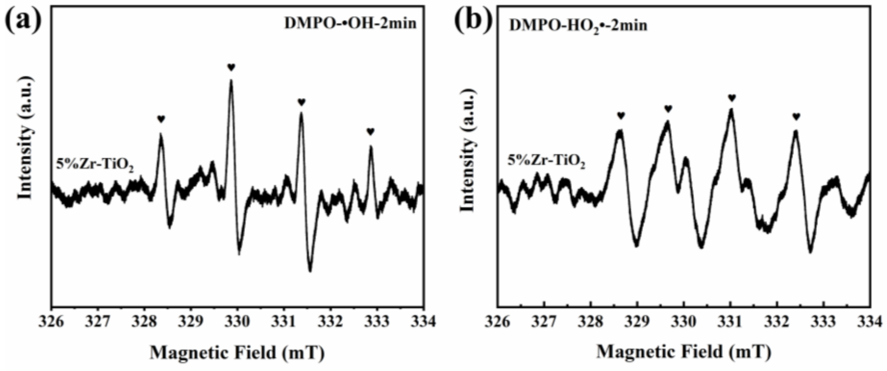

Figure S3. EPR spectra of 5%Zr-TiO_2.

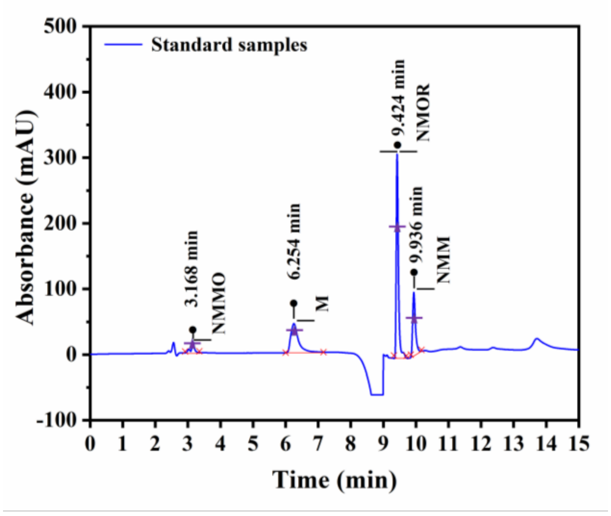

Figure S4. HPLC standard curve.

Table S2. Compared with reported catalyst properties.

Catalysts	Conditions	Yield %	References
NH_4HCO_3	$[H_2O_2]/[NMM]/[HCO_3^-]$ = 5:1:2.5, 25 °C, 1 h	93.0	[9]
TS-1	$[H_2O_2]/[NMM]$ = 1.61:1.3, 70 °C, 6h, 5%	89.2	[10]
5%Zr-TiO_2	$[H_2O_2]/[NMM]$ = 1.3:1, 40 °C, 3 h, 2‰	97.6	this research
